# Platelet-Rich Plasma Proteome of Mares Susceptible to Persistent-Breeding-Induced Endometritis Differs from Resistant Mares

**DOI:** 10.3390/ani14142100

**Published:** 2024-07-18

**Authors:** Guilherme Novello, Fabiana F. Souza, Igor F. Canisso

**Affiliations:** 1 Department of Veterinary Surgery and Animal Reproduction, School of Veterinary Medicine and Animal Science, Sao Paulo State University (UNESP), Botucatu 01419-901, SP, Brazil; guilhermenovello@outlook.com (G.N.); fafesouza@fmvz.unesp.br (F.F.S.); 2 Department of Veterinary Clinical Medicine, University of Illinois Urbana-Champaign, Urbana, IL 61822, USA

**Keywords:** protein, inflammation, equine

## Abstract

**Simple Summary:**

Mares are classified as susceptible or resistant to PBIE based on their susceptibility to this condition. Persistent-breeding-induced endometritis (PBIE) is an important cause of poor fertility in mares. Uterine infusion of platelet-rich plasma (PRP) reduces inflammation and infections associated with PBIE. However, the protein composition of PRP in mares, particularly those susceptible to PBIE, remains unknown. This study characterized the protein composition of PRP in mares susceptible and resistant to PBIE. The PRP proteome varies between these two categories. The results could suggest mares susceptible to PBIE could benefit from PRP prepared from mares resistant to PBIE, potentially improving their fertility.

**Abstract:**

Persistent-breeding-induced endometritis (PBIE) is the leading cause of subfertility and poor reproductive efficiency in mares. Platelet-rich plasma (PRP) treatment has been shown to mitigate PBIE, reduce uterine infections, and improve fertility in mares. However, the proteome of PRP in mares, particularly those susceptible to PBIE, remains unknown. This study aimed to fill this knowledge gap by comparing the most abundant proteins present in PRP prepared from mares with histories of being susceptible or resistant to PBIE. The study involved twelve light-breed mares: seven susceptible and five resistant to PBIE. A complete blood count and physical examination were performed on each mare before blood drawing to ensure good health. The PRP was prepared following collection in a blood transfusion bag and double centrifugation. Platelet counts in the PRP were compared across the groups. The PRP was cryopreserved in liquid nitrogen until proteomics could be completed. Physical parameters and complete blood cell counts were within normal ranges. The platelet counts for resistant (561 ± 152 × 10^3^) and susceptible mares (768 ± 395 × 10^3^) differed (*p* < 0.05). One hundred and five proteins were detected in all mares, and four proteins were more abundant in resistant mares (*p* < 0.05). The proteins were apolipoprotein C-II, serpin family G member 1, protection of telomeres protein 1, and non-specific serine/threonine protein kinase. All these proteins are linked to the immune response. These results suggest that PRP prepared from mares resistant to PBIE may be more beneficial in mitigating PBIE in mares, offering a promising avenue for improving equine reproductive health. However, this remains to be determined with *in vivo* studies.

## 1. Introduction

After mating or artificial insemination, post-breeding endometritis is a physiological process that clears excess semen, debris, and microorganisms from the uterine lumen [1]. However, when the inflammation persists, this is deemed persistent breeding-induced endometritis (PBIE) [2]. Mares suffering from PBIE have reduced pregnancy rates and poor reproductive efficiency, negatively impacting the horse breeding industry [3]. This issue is of a significant concern, as endometritis was the third most common problem encountered in clinical practice by equine practitioners in one study [4]. Poor fertility in mares susceptible to PBIE is due to a hostile uterine environment upon the embryo’s arrival in the uterus, precisely due to an excess of inflammatory products and mediators in the uterine fluid [5,6,7,8]. Mares susceptible to PBIE have a pronounced expression of proinflammatory cytokines and reduced anti-inflammatory cytokines to regulate acute inflammation, compared to resistant mares [9,10,11].

Most mares suffering from PBIE respond to traditional therapy involving uterine lavage, ecbolic, and steroidal and non-steroidal anti-inflammatory drugs, uterine infusions, or systemic administrations of antibiotics [12,13,14,15]. However, some mares do not respond to traditional therapy for PBIE [7]. Recent studies have been performed to develop alternative treatments to antibiotics; these include acetylcysteine, hydrogen peroxide, dimethyl-sulfoxide, iodine solution, kerosene, hyper-immune plasma, blood plasma, mycobacterium cell wall extract, stem cells and their by-products, and platelet-rich plasma (PRP) [12,13,14,15].

Platelet-rich plasma is gaining popularity in clinical practice for its anti-inflammatory and natural antimicrobial properties. It consists of whole blood plasma with a high platelet concentration (i.e., 3–5-fold higher), and it contains diverse growth factors that can act in injured tissue by inhibiting NF-kB and downregulating proinflammatory cytokines in the mare endometrium [12,13]. The treatment of subfertile susceptible mares with PRP can mitigate PBIE, reduce uterine infections, and improve fertility [16,17,18,19,20]. Despite the potential of equine PRP, the proteome has not been studied. This study, therefore, fills a significant gap in the literature by comparing the most abundant proteins present in PRP prepared from mares with histories of being susceptible or resistant to PBIE.

## 2. Materials and Methods

### 2.1. Animals and Husbandry

Twelve clinically healthy mares aged between 3 and 23 years (susceptible group: 6, 6, 7, 8, 9, 21, and 23 years old; resistant group: 3, 4, 5, 8, and 20 years old) were enrolled in the study. All mares were housed in a privately owned breeding center in Passo Fundo, Rio Grande do Sul, Brazil. The mares were maintained on pasture and supplemented with ryegrass hay water and trace minerals ad libitum. Based on their past breeding history, mares were classified as susceptible (*n* = 7) or resistant (*n* = 5) to PBIE. Mares susceptible to endometritis had a history of uterine fluid accumulation, repeated uterine infections post-breeding, and poor conception rates. In contrast, those resistant to endometritis were fertile mares with no history of intrauterine fluid accumulations or uterine infections after breeding.

The day before the blood sampling, mares had a complete blood cell count and full physical examination to determine their health statuses. All mares had physical parameters and complete blood cell counts within normal limits. 

### 2.2. Platelet-Rich Preparation

The PRP was prepared as previously described elsewhere [21]. This involved blood harvesting from each animal by venipuncture of the jugular vein using an 18G needle into a blood transfusion bag (Jorgensen Labs, Loveland, CO, USA) containing 63 mL of citrate-phosphate-dextrose solution with adenine as an anticoagulant. Four hundred milliliters of whole blood were split into eight 50 mL tubes, and the samples were centrifuged at 400× *g* for 15 min. The supernatant was transferred into 15 mL conical tubes and centrifuged at 1000× *g* for 10 min. After the second centrifugation, 2.5 mL of plasma at the bottom of each tube was deemed as PRP. Platelet, white blood, and red blood cell concentrations were determined in the whole blood and PRP samples using manual counting with a hemocytometer at a privately owned Clinical Pathology Laboratory (Diavet Veterinary Diagnostic Laboratory, Passo Fundo, Rio Grande do Sul, Brazil). After centrifugation, 5 mL of PRP was stored in liquid nitrogen at −196 °C and transported to the Sao Paulo State University (UNESP) Proteomics Lab, Botucatu.

### 2.3. Extraction of PRP Proteins

The frozen PRP samples were taken from liquid nitrogen and thawed in an ice bath. Then, RIPA solution (150 mmol NaCl, 1% Triton X-10, 1% sodium deoxycholate, 0.1% SDS, and 50 mmol TRIS–HCL, pH 7.5) and protease inhibitors (1.0 µg/mL aprotinin, 1.0 µg/mL leupeptin, 1.0 µg/mL phenylmethylsulphonyl fluoride [PMSF], and 10 µg/mL EDTA) were added. The samples were sonicated using a 3 mm probe at 20% amplitude for 30 s in an ice bath. Three sonication series were performed on each sample, with a 1 min interval between the series. Then, the samples were centrifuged at 10,000× *g* at 4 °C for 30 min. The total protein concentration was determined on a nano spectrophotometer (NanoDrop A280, Thermo Scientific NanoDrop One Microvolume UV-VIS spectrophotometers, Waltham, MA, USA) at an absorbance of 280 nm (A280).

### 2.4. In-Gel Tryptic Digestion of Samples

Samples were subjected to SDS-PAGE using 50 µg of total protein and a 10% separating gel. One of the wells was filled with 50 µg of bovine serum albumin as a protein quantity standard. The run was stopped when the samples reached the separation gel. Then, the gel was stained with colloidal Coomassie blue [22,23]. As previously described, bands were cut and prepared for mass spectrometry [24]. The gel fragments were de-stained five times with 50% acetonitrile in 25 mmol of ammonium bicarbonate buffer (AmBic) and dehydrated in 100% acetonitrile. A solution of 20 mmol DTT in 50 mmol AmBic was used for reduction, followed by alkylation with 55 mmol iodoacetamide in 50 mmol AmBic. Digestion was conducted with trypsin (1:25 substrate/enzyme; Code No V511, Promega Corporation, Madison, WI, USA) for 14 h. Trypsin action was blocked with 5% formic acid in 50% acetonitrile/50 mmol AmBic. Peptides were eluted thrice: (1) 1% formic acid in 60% methanol; (2) 1% formic acid in 50% acetonitrile/ultrapure water; (3) acetonitrile 100%. The entire volume recovered in each elution was deposited in the same tube and reduced to ~1 µL (Speed Vac™, SPD1010 Integrated SpeedVac™ Systems, Thermo Fisher Scientific Inc., Waltham, MA, USA). The samples were then stored at −20 °C until mass spectrometry could be completed.

### 2.5. Mass Spectrometry

For mass spectrometry, samples were thawed, diluted in 0.1% formic acid (0.7 μg protein/μL), homogenized, and centrifuged at 1100× *g* for 5 min. Then, 15 μL of the supernatant was deposited in glass tubes for mass spectrometry (Clear glass 12 × 32 mm, Waters Corporation, Milford, MA, USA). An aliquot (4.5 μL) was separated on a C18 nano-chromatography column (100 μm × 100 mm; nano ACQUITY UPLC^®^, Waters Corporation, Milford, MA, USA) coupled to the Q-Tof mass spectrometer (Micromass^®^ Q-Tof PREMIER^®^ Mass 12 Spectrometer, Waters Corporation, Milford, MA, USA), which contained an electrospray ionization source with a flow rate of 0.60 μL/minute, a voltage of 3.5 kV, a voltage cone of 30 V, and a temperature of 100 °C. The mobile-phase gradient was 2–90% acetonitrile in 0.1% formic acid for 45 min. The equipment was operated in the top three modes, and MS spectra were acquired, followed by MS/MS of the three most intense peaks detected. After MS/MS fragmentation, ions were kept on an exclusion list for 60 s. The searching parameters included trypsin as the protease, one allowed cleavage, cysteine carboxyamidomethylation as a fixed modification, methionine oxidation as a variable modification, 1 Da error tolerance for the mass spectrum (MS), and the mass of the fragment ions (MS/MS) and the monoisotopic molecular weight.

### 2.6. Acquisition of Proteomics Data

Raw data were converted to a peak list (.mfg) without adding scans, and spectra were acquired using Mass Lynx™ v.4.1 software (Waters Corporation, Milford, MA, USA). The analysis was performed in Mascot Distiller 2.4.0.0 software (Matrix Science Inc., Boston, MA, USA) to obtain a relative quantification of each protein in the mixture using an exponentially modified protein abundance index (emPAI). Searching of the results was performed using the taxonomy *Equus caballus* (UP000002281) from the UniProt KB database (www.uniprot.org/ accessed on 1 April 2024). Mass spectra and protein identification results were validated using Protein Pilot 4.0 software (AB Sciex, Framingham, MA, USA).

### 2.7. Statistical Analysis

Data were normalized to obtain accurate estimates of the biological effects and remove outliers from the experimental variation. For this, proteins present in at least 50% of the samples in each group were considered for analysis. Outliers were corrected by dividing the value of each protein by the sum of the same across all groups. Univariate (*t*-test, volcano plot, and fold change) and multivariate analyses (non-hierarchical grouping: principal component analysis [PCA], partial least squares discriminant analysis [PLS-DA], dendrogram and heatmap, sensitivity and specificity of proteins found using ROC [receiver operator characteristic] curves) were performed. The results were significant when the false discovery rate (FDR) < 0.05. The analyses were conducted at MetaboAnalyst [23]. Pathway enrichment in Gene Ontology was obtained using ShinyGO 0.77 software (http://bioinformatics.sdstate.edu/go/, accessed on 1 May 2024). The relationships of proteins between the groups are represented by a Venn diagram (https://bioinformatics.psb.ugent.be/webtools/Venn/, accessed on 1 May 2024).

## 3. Results

**Figure 1:**
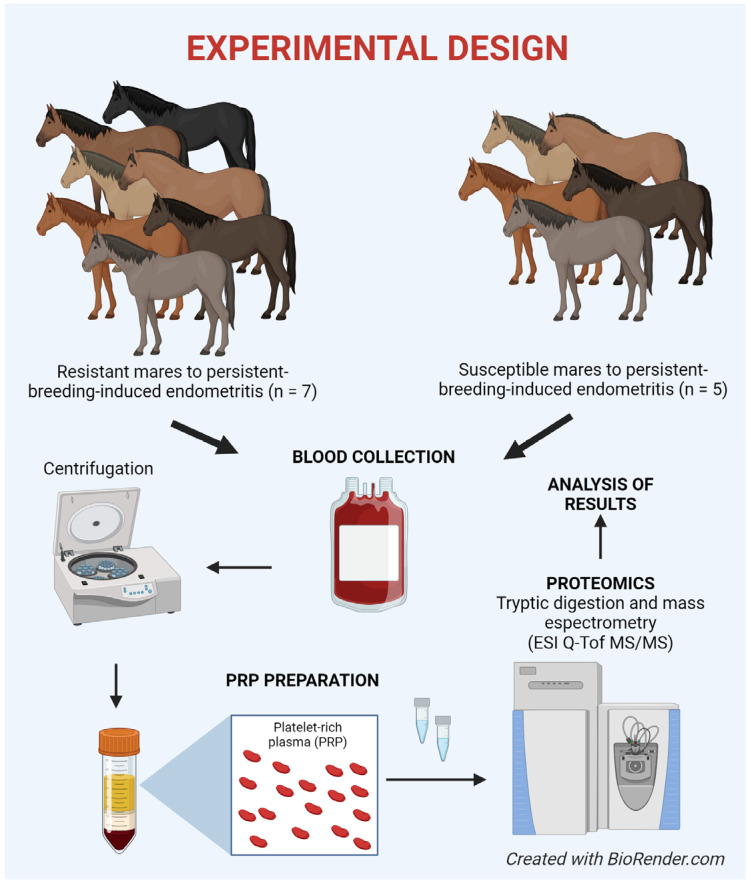
Experimental design.

The platelet counts for the resistant (561 ± 152 × 10^3^) and susceptible mares (768 ± 395 × 10^3^) were different (*p* < 0.05) (Table 1). One hundred and five unique proteins were found, and five proteins were found to be overly abundant in the PRP of mares resistant to PBIE (Figure 1). The list of proteins is depicted in Tables S1 and S2.

**Figure 2:**
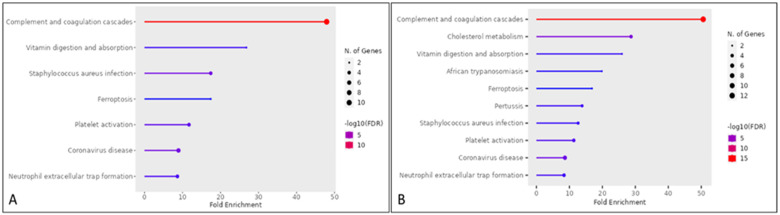
Enriched Gene Ontology pathways from proteomics of platelet-rich plasmas from mares susceptible (**A**) and resistant (**B**) to persistent-breeding-induced endometritis (http://bioinformatics.sdstate.edu/go/).

**Figure 3:**
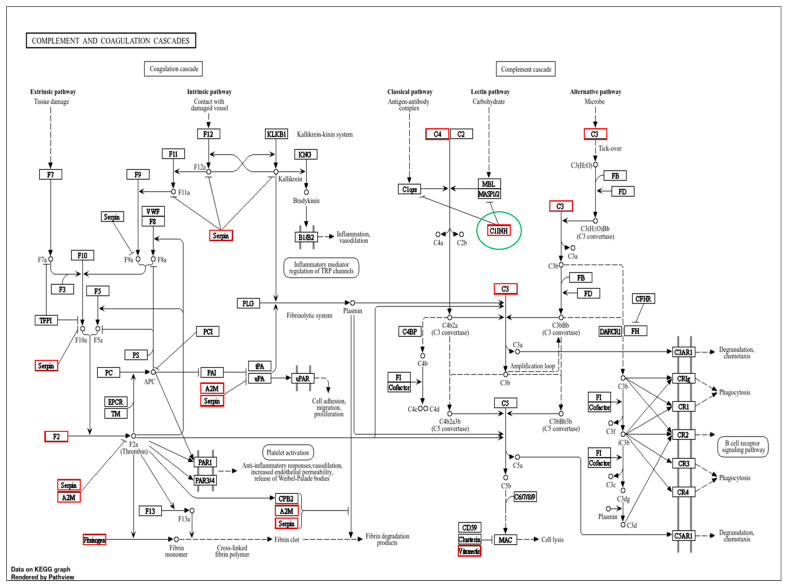
The PRP Gene Ontology prepared from the blood of mares susceptible and resistant to endometritis showed an enhanced complement and coagulation cascade pathway. The highlighted green circle indicates the only difference between the groups in this evaluation and corresponds to the PRP of resistant mares. The red boxes show major players.

**Figure 4:**
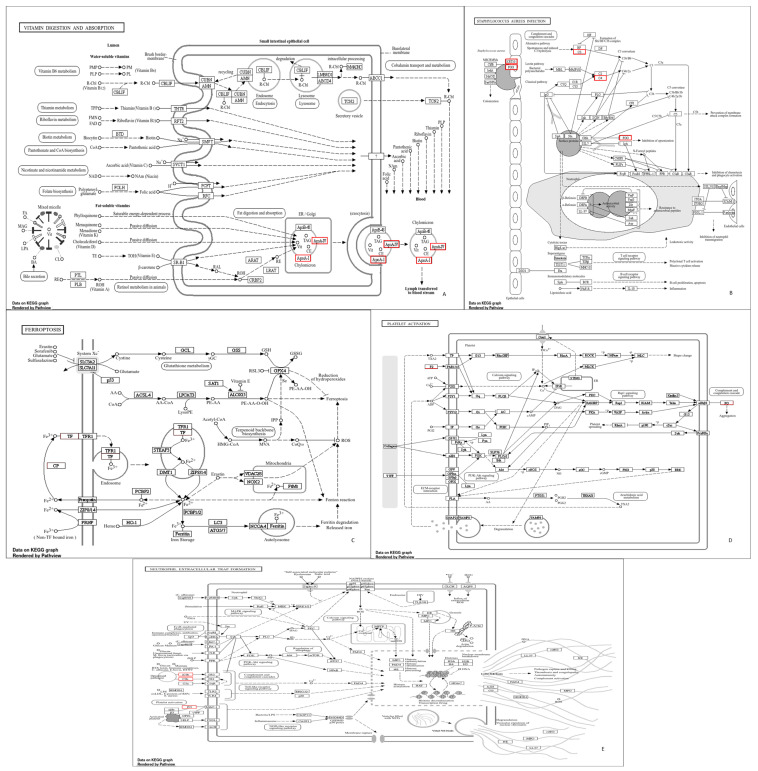
Enriched pathways found in Gene Ontology are common in PRP prepared from the blood of mares susceptible and resistant to endometritis. Vitamin digestion and absorption (**A**), Staphylococcus aureus infection (**B**), ferroptosis (**C**), platelet activation (**D**), and neutrophil extracellular trap formation (**E**) are shown.

The gene ontology enrichment in the susceptible and resistant mares was similar between the groups (Figure 2). However, the other three enriched pathways were found in the PRP of the mares resistant to PBIE; therefore, seven pathways were found in susceptible mares, whereas ten were found in resistant mares. These pathways directly affect the immune system [24,25,26]. Furthermore, it was verified that in the complement and coagulation cascade pathways, a different gene was expressed between the groups (Figure 3). The common pathways between the groups (with no differentially detected genes) are represented in Figure 4.

**Figure 5:**
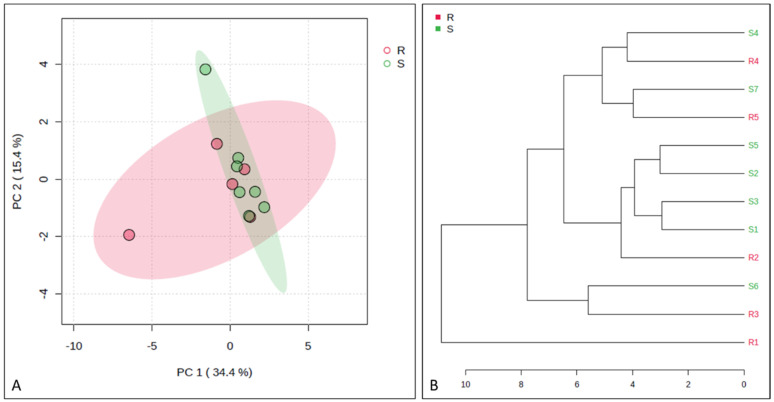
Principal component analysis (PCA; (**A**)) and dendrogram (**B**). Note an overposition in the PCA; a similar result was observed in the dendrogram. PC1 + PC2 = 49.8%.

**Figure 6:**
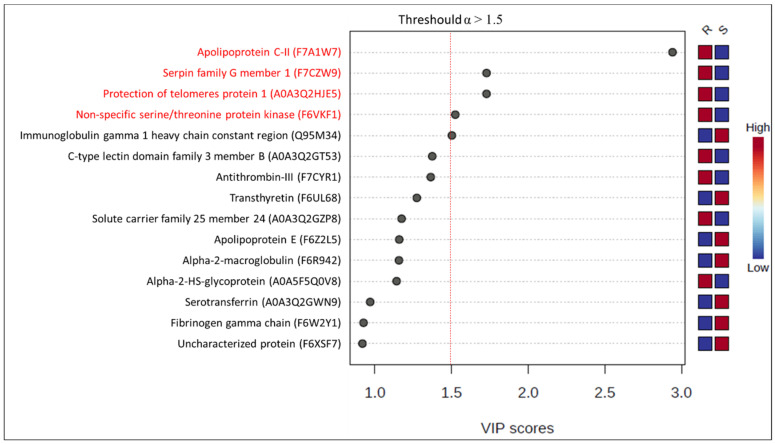
VIP score resulting from PLS-DA comparing the most abundant proteins in the PRP of mares susceptible (S) and resistant (R) to persistent-breeding-induced endometritis. Proteins highlighted in red were found to be differentially abundant in the *t*-test (*p* < 0.05). Protection of telomere protein 1 was the only protein with an AUC > 0.80 in the ROC curve (Figure 7).

**Figure 7:**
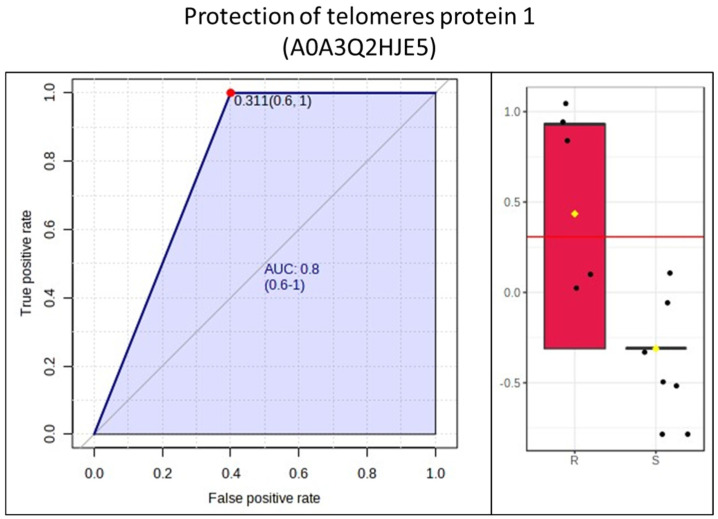
Receiving operating curve of protection of telomeres protein 1. R = resistant; S = susceptible. The dots denote outliers.

Figure 5 represents the PCA and dendrogram, in which a smooth separation between the groups can be observed, as the sum of the main components was 49.8%. Four proteins (apolipoprotein C-II; serpin family G member 1; protection of telomeres protein 1; non-specific serine/threonine protein kinase) were found to be differentially abundant in mares resistant to endometritis in the VIP score (α > 1.5) and in the *t*-test (*p* < 0.05). In contrast, immunoglobin gamma 1 heavy-chain constant region was more abundant in mares susceptible to endometritis in the VIP score (α > 1.5) (Figure 6). Protection of telomers protein 1 was overly abundant in mares resistant to endometritis compared to susceptible mares (Figure 7).

## 4. Discussion

This study set forth to compare the most abundant proteins present in PRP prepared from mares with histories of being susceptible or resistant to PBIE. The proteins found with different abundance between the groups were related to the immune system. C1 inhibitor (C1-inh, C1 esterase inhibitor), which is enriched in endometritis-resistant mares, is a protease inhibitor belonging to the serpin superfamily [27]. It modulates one of the endometritis activation pathway complement systems, and its function is to inhibit the complement system in the classical and lecithin pathways [28]. It also inhibits proteases in the fibrinolytic, coagulation, and kinin pathways [29]. One of the proteins related to this pathway was the serpin family G member 1, part of a superfamily of proteins associated with the immune system. Among its different functions, the protein is involved in the negative regulation of complement system activation (UniProt, https://www.uniprot.org/), which may also justify the greater abundance of these macromolecules in mares resistant to post-breeding endometritis.

Apolipoprotein C-II (apoC-II) is a protein found in triglyceride-rich lipoproteins during fasting [30]. In macrophages, however, the main transcription factors for the APOC2 gene include the liver X receptor (LXR), whose role is to correct cellular cholesterol [31,32], and the signal transducer and activator of transcription 1 (STAT1), which mediates cellular responses to interferons and specific cytokines and growth factors [33]. LXR has an anti-inflammatory role in macrophages [32]. Therefore, increased inflammation can inhibit the inflammatory response that occurs in mares that are resistant to endometritis.

Protection of telomere protein 1 (POT1) is part of a protein complex called shelterin proteins, which prevent the recognition of chromosomal ends containing DNA breaks, preventing their degradation. Furthermore, these proteins are considered to be a response pathway to DNA damage. These functions can also be performed in cells of the immune system [34], given that during chronic inflammation, there is a reduction in the size of the telomeres in leukocytes [35]. In humans, telomere shortening occurs in conjunction with normal aging [36]. However, inflammation, oxidative stress, and other genotoxic stressors also increase the rate of telomere wear [37]. Cellular senescence induces the loss of proliferative capacity, but senescent cells are still metabolically active with altered secretory phenotypes. This change increases the production of inflammatory cytokines for chronic inflammation [38,39]. Furthermore, telomere shortening in lymphocytes is associated with the dysregulation of immune homeostasis [40]. Since POT1 is part of this system and is in lower abundance in susceptible mares, it is possible to suggest that PRP in these mares may not contribute adequately to the moderation of uterine inflammation; it could also explain the chronic uterine inflammation associated with elderly mares. Interestingly, this was the only protein with an AUC (ROC curve) > 0.80 found in resistant mares.

Non-specific serine/threonine protein kinase is part of a large family of proteins. It is involved in cell proliferation, differentiation, survival, and cellular responses, and it plays a vital role in signal transduction pathways, including signaling and responses to thermal stress in plants [41]. Although we did not find a relationship with the inflammatory process, its functions are related to this event, which could justify the moderation of the response in resistant mares.

The C4a anaphylatoxin protein produced when the complement system is activated is one of more than forty proteins in this group [42] and has an antibacterial effect [43]. However, its anti-inflammatory effect is somewhat questionable [42]. Proteins such as alpha-2-HS-glycoprotein have several functions, some of which are inflammatory mediators and anti-inflammatory partners [44], functions not found in C4a. Another complement system protein was found in C3, a protein that releases peptides, such as C3a, and exerts multiple proinflammatory functions, involving histamine release from mast cells, smooth muscle contraction, and increased vascular permeability [45], in addition to exercising a strong antibacterial function [46]. Given this, it is essential to highlight that the three proteins mentioned above were found in the group of mares resistant to endometritis. This fact leads us to believe these proteins help clean the uterus with their most diverse functions.

Controversy exists about whether immunoglobulin concentrations differ in the uterine fluid of mares susceptible or resistant to PBIE. One study found no difference in immunoglobulin concentration in the uterine fluid between resistant and susceptible mares [47]. In contrast, another study showed that susceptible mares presented higher amounts of IgA, IgG, and IgG(T) in their uterine secretions [48]. In the present study, immunoglobin gamma 1 heavy-chain constant region was upregulated in mares susceptible to endometritis in terms of VIP score (α > 1.5). It remains to be determined if these higher concentrations have any role in treating PRP in susceptible mares. 

The method used herein to prepare PRP followed a standard method used in the author’s clinical practice and in studies of the principal investigator [49]. The results obtained herein have direct application to clinical practice. While it remains to be determined if there are benefits of using the plasma of resistant mares rather than the plasma of susceptible mares, the difference in the proteome between these two categories is certainly encouraging for further studies and use in clinical practice.

## 5. Conclusions

In conclusion, most of the proteins found in platelet-rich plasma are the same for mares resistant and susceptible to persistent-breeding-induced endometritis; despite this, four proteins related to the immune response were found on a large scale in resistant mares, suggesting that plasma from resistant mares could be beneficial for mares susceptible to PBIE. These results indicate that PRP prepared from mares resistant to PBIE may be more useful in mitigating PBIE in mares, and that homologous PRP could be superior to autologous; however, this remains to be determined.

## Figures and Tables

**Table 1:** Platelets, white blood cells (WBCs), and red blood cells (RBCs) in the whole blood and platelet-rich plasma (PRP) of mares resistant and susceptible to persistent breeding-induced endometritis.

	Whole Blood			PRP		
	Platelets (×10^3^)	WBCs (×10^3^)	RBCs (×10^6^)	Platelets (×10^3^)	WBCs (×10^3^)	RBCs (×10^6^)
Resistant (*n* = 5)	163 ± 32.2	7.4 ± 1.8	931 ± 436	561 ± 152	12.6 ± 4.45	3.40 ± 4.92
Susceptible (*n* = 7)	168 ± 32.6	9.3 ± 1.9	889 ± 94	768 ± 395	17.7 ± 17.9	4 ± 3.95

Platelet-rich plasma (PRP); white blood cells (WBCs); red blood cells (RDBs).

## Data Availability

Data are contained within the article.

## Supplementary Materials

The following supporting information can be downloaded at https://www.mdpi.com/article/10.3390/ani14142100/s1, Table S1: Protein information on PRP from mares susceptible to persistent-breeding-induced endometritis (www.uniprot.gov); Table S2: PRP protein information on mares resistant to post-breeding endometritis (www.uniprot.gov).
