# Supplementary material for: Platelet-Rich Plasma Proteome of Mares Susceptible to Persistent-Breeding-Induced Endometritis Differs from Resistant Mares
Source: Animals (Basel). 2024 Jul 18;14(14):2100. doi: 10.3390/ani14142100 (PMC11273647; doi:10.3390/ani14142100)
Supplement: Supplementary file 1 [file animals-14-02100-s001.zip › animals-3008356-supplementary.pdf]

S-Table 1. Protein information of PRP from sensible mares to post-breeding endometritis (www.uniprot.gov).

UniProt ID	Protein name	Gene Name	Gene Ontology (biological process)	Gene Ontology (cellular component)	Gene Ontology (molecular function)
A0A3Q2H333	Albumin	ALB		Extracellular space [GO:0005615]	Metal ion binding [GO:0046872]
P35747	Albumin (allergen Equ c 3)	ALB	Cellular response to calcium ion starvation [GO:0072732]; cellular response to starvation [GO:0009267]; negative regulation of mitochondrial depolarization [GO:0051902]	Cytoplasm [GO:0005737]; extracellular space [GO:0005615]; protein-containing complex [GO:0032991]	DNA binding [GO:0003677]; enterobactin binding [GO:1903981]; fatty acid binding [GO:0005504]; metal ion binding [GO:0046872]; pyridoxal phosphate binding [GO:0030170]; toxic substance binding [GO:0015643]
A0A5F5PYW9<br>A0A5F5PSR8<br>A0A3Q2H846<br>F7CSL8<br>F7CYP1	Alpha-1-antitrypsin	SPI2		Extracellular space [GO:0005615]	Serine-type endopeptidase inhibitor activity [GO:0004867]
B5BV00	Alpha-1-antitrypsin	Spi2-1		Extracellular space [GO:0005615]	Serine-type endopeptidase inhibitor activity [GO:0004867]
B5BV07	Alpha-1-antitrypsin	Spi2-8		Extracellular space [GO:0005615]	Serine-type endopeptidase inhibitor activity [GO:0004867]
B5BV09	Alpha-1-antitrypsin	Spi2-10		Extracellular space [GO:0005615]	Serine-type endopeptidase inhibitor activity [GO:0004867]
B5BV12	Alpha-1-antitrypsin	Spi2-13		Extracellular space [GO:0005615]	Serine-type endopeptidase inhibitor activity [GO:0004867]
B5BV13	Alpha-1-antitrypsin	Spi2-14		Extracellular space [GO:0005615]	Serine-type endopeptidase inhibitor activity [GO:0004867]
F6VJR6	Alpha-1-B glycoprotein	A1BG		Extracellular region [GO:0005576]; plasma membrane [GO:0005886]	
F7C450<br>A0A5F5Q0V8<br>A0A5F5PJ36	Alpha-2-HS-glycoprotein (Fetuin-A)	AHSG	Acute-phase response [GO:0006953]; negative regulation of bone mineralization [GO:0030502]; ossification [GO:0001503]; positive regulation of phagocytosis [GO:0050766]; regulation of inflammatory response [GO:0050727]	Extracellular matrix [GO:0031012]; extracellular region [GO:0005576]; extracellular space [GO:0005615]; Golgi apparatus [GO:0005794]	Cysteine-type endopeptidase inhibitor activity [GO:0004869]; endopeptidase inhibitor activity [GO:0004866]
F6RI47<br>F6R942	Alpha-2-macroglobulin	A2M	Negative regulation of complement activation, lectin pathway [GO:0001869]	Extracellular space [GO:0005615]	Calcium-dependent protein binding [GO:0048306]; interleukin-1 binding [GO:0019966]; interleukin-8 binding [GO:0019959]; protease binding [GO:0002020]; serine-type endopeptidase inhibitor activity [GO:0004867]; signaling receptor binding [GO:0005102]; tumor necrosis factor binding [GO:0043120]
A0A5F5PSV7	Angiotensinogen (Serpin A8)	AGT	Regulation of systemic arterial blood pressure by renin-angiotensin [GO:0003081]; vasoconstriction [GO:0042310]	Extracellular space [GO:0005615]	Serine-type endopeptidase inhibitor activity [GO:0004867]
F7CYR1	Antithrombin-III (Serpin C1)	SERPINC1	Blood coagulation [GO:0007596]; regulation of blood coagulation [GO:0030193]	Extracellular space [GO:0005615]	Heparin binding [GO:0008201]; serine-type endopeptidase inhibitor activity [GO:0004867]
F6Z2L5	Apolipoprotein A-I (Apolipoprotein A1)	APOA1	Lipid transport [GO:0006869]; lipoprotein metabolic process [GO:0042157]; phosphatidylcholine metabolic process	Chylomicron [GO:0042627]; high-density lipoprotein particle [GO:0034364]; multivesicular body [GO:0005771]; very-	Heparin binding [GO:0008201]; high-density lipoprotein particle receptor binding [GO:0070653]; lipid binding [GO:0008289]

			[GO:0046470]; regulation of intestinal cholesterol absorption [GO:0030300]	low-density lipoprotein particle [GO:0034361]	
A0A5F5PEE6	Apolipoprotein A-IV (Apolipoprotein A4)	APOA4	Cholesterol efflux [GO:0033344]; cholesterol homeostasis [GO:0042632]; lipoprotein metabolic process [GO:0042157]; phosphatidylcholine metabolic process [GO:0046470]; phospholipid efflux [GO:0033700]; positive regulation of fatty acid biosynthetic process [GO:0045723]; positive regulation of triglyceride catabolic process [GO:0010898]; regulation of intestinal cholesterol absorption [GO:0030300]; reverse cholesterol transport [GO:0043691]; triglyceride homeostasis [GO:0070328]; very-low-density lipoprotein particle remodeling [GO:0034372]	Chylomicron [GO:0042627]; high-density lipoprotein particle [GO:0034364]; multivesicular body [GO:0005771]; very-low-density lipoprotein particle [GO:0034361]	Heparin binding [GO:0008201]; phosphatidylcholine binding [GO:0031210]; phosphatidylcholine-sterol O-acyltransferase activator activity [GO:0060228]
F6XSF7	C4a anaphylatoxin	LOC1000592 39	Complement activation [GO:0006956]; complement activation, classical pathway [GO:0006958]; inflammatory response [GO:0006954]; innate immune response [GO:0045087]	Extracellular space [GO:0005615]	Endopeptidase inhibitor activity [GO:0004866]
F6PQ46	Ceruloplasmin	CP	Copper ion transport [GO:0006825]; iron ion transport [GO:0006826]	Extracellular space [GO:0005615]; plasma membrane [GO:0005886]	Copper ion binding [GO:0005507]; oxidoreductase activity [GO:0016491]; protein-folding chaperone binding [GO:0051087]
A0A3Q2L3I3	Clusterin	CLU		Chromaffin granule [GO:0042583]; cytosol [GO:0005829]; endoplasmic reticulum [GO:0005783]; extracellular region [GO:0005576]; mitochondrial membrane [GO:0031966]; nucleus [GO:0005634]; perinuclear region of cytoplasm [GO:0048471]	
A0A3Q2HWQ6	Complement C3	C3	Complement activation [GO:0006956]; complement activation, alternative pathway [GO:0006957]; complement activation, classical pathway [GO:0006958]; inflammatory response [GO:0006954]	Extracellular space [GO:0005615]	Endopeptidase inhibitor activity [GO:0004866]
A0A3Q2GT53	C-type lectin domain family 3 member B	CLEC3B	Bone mineralization [GO:0030282]; cellular response to organic substance [GO:0071310]; cellular response to transforming growth factor beta stimulus [GO:0071560]; ossification [GO:0001503]	Cytoplasm [GO:0005737]; extracellular space [GO:0005615]; granular component [GO:0001652]	Calcium ion binding [GO:0005509]; heparin binding [GO:0008201]; kringle domain binding [GO:0036143]
A0A5F5PFU8	Enhancer of polycomb homolog 1	EPC1	Regulation of transcription by RNA polymerase II [GO:0006357]	Piccolo nua4 histone acetyltransferase complex [GO:0032777]	
A0A3Q2HAZ4	Exocyst complex component	EXOC6	Intracellular protein transport [GO:0006886]; vesicle tethering involved in exocytosis [GO:0090522]	Exocyst [GO:0000145]	
A0A3Q2LTU8	Ferric oxidoreductase domain-containing protein	STEAP1		Membrane [GO:0016020]	
F6RRV1 A0A5F5PUG6	Fetuin B	FETUB	Binding of sperm to zona pellucida [GO:0007339]	Extracellular space [GO:0005615]	Cysteine-type endopeptidase inhibitor activity [GO:0004869]; metalloendopeptidase inhibitor activity [GO:0008191]

A0A3Q2HTG2	Fibrinogen alpha chain	FGA	Blood coagulation, common pathway [GO:0072377]; cell-matrix adhesion [GO:0007160]; fibrinolysis [GO:0042730]; induction of bacterial agglutination [GO:0043152]; negative regulation of endothelial cell apoptotic process [GO:2000352]; negative regulation of extrinsic apoptotic signaling pathway via death domain receptors [GO:1902042]; plasminogen activation [GO:0031639]; platelet aggregation [GO:0070527]; positive regulation of ERK1 and ERK2 cascade [GO:0070374]; positive regulation of exocytosis [GO:0045921]; positive regulation of heterotypic cell-cell adhesion [GO:0034116]; positive regulation of peptide hormone secretion [GO:0090277]; positive regulation of protein secretion [GO:0050714]; positive regulation of vasoconstriction [GO:0045907]; protein polymerization [GO:0051258]; response to calcium ion [GO:0051592]	Endoplasmic reticulum [GO:0005783]; external side of plasma membrane [GO:0009897]; fibrinogen complex [GO:0005577]; platelet alpha granule [GO:0031091]	Cell adhesion molecule binding [GO:0050839]; signaling receptor binding [GO:0005102]; structural molecule activity [GO:0005198]
P14452	Fibrinogen alpha chain [Cleaved into: Fibrinopeptide A]	FGA	Adaptive immune response [GO:0002250]; blood coagulation [GO:0007596]; innate immune response [GO:0045087]	Extracellular region [GO:0005576]	
A0A5F5PJQ9	Fibrinogen beta chain	FGB	Platelet activation [GO:0030168]; protein polymerization [GO:0051258]	Fibrinogen complex [GO:0005577]	Signaling receptor binding [GO:0005102]
F6YW53	Geminin DNA replication inhibitor	GMNN	Animal organ morphogenesis [GO:0009887]; DNA replication preinitiation complex assembly [GO:0071163]; negative regulation of cell cycle [GO:0045786]; negative regulation of DNA replication [GO:0008156]; negative regulation of DNA-templated DNA replication [GO:2000104]; regulation of DNA-templated DNA replication initiation [GO:0030174]; regulation of mitotic cell cycle [GO:0007346]	Cytosol [GO:0005829]; nucleoplasm [GO:0005654]; nucleus [GO:0005634]; transcription repressor complex [GO:0017053]	Chromatin binding [GO:0003682]; DNA-binding transcription factor binding [GO:0140297]; histone deacetylase binding [GO:0042826]; transcription corepressor activity [GO:0003714]
A0A3Q2HBR4 A0A5F5PPX0	Haptoglobin	LOC100067869	Acute-phase response [GO:0006953]; defense response to bacterium [GO:0042742]; proteolysis [GO:0006508]	Extracellular region [GO:0005576]	Antioxidant activity [GO:0016209]; hemoglobin binding [GO:0030492]; serine-type endopeptidase activity [GO:0004252]
F7C3F9	Hemicentin 2	HMCN2	Homophilic cell adhesion via plasma membrane adhesion molecules [GO:0007156]	Basement membrane [GO:0005604]; cell cortex [GO:0005938]; cell junction [GO:0030054]; extracellular region [GO:0005576]	Calcium ion binding [GO:0005509]
F6X1I8	Hemopexin	HPX	Heme metabolic process [GO:0042168]; hemoglobin metabolic process [GO:0020027]; intracellular iron ion homeostasis [GO:0006879]; positive regulation of humoral immune response mediated by circulating immunoglobulin [GO:0002925]; positive regulation of immunoglobulin production [GO:0002639]; positive regulation of type II interferon-mediated signaling pathway [GO:0060335]; positive	Extracellular space [GO:0005615]	Heme transmembrane transporter activity [GO:0015232]; metal ion binding [GO:0046872]

			regulation of tyrosine phosphorylation of STAT protein [GO:0042531]; type II interferon-mediated signaling pathway [GO:0060333]		
A0A915VF08	Histidine-rich glycoprotein (Histidine-proline-rich glycoprotein)	hrg			Cysteine-type endopeptidase inhibitor activity [GO:0004869]
A0A3Q2I554	Histone acetyltransferase (EC 2.3.1.48)	KAT5	Positive regulation of DNA-templated transcription [GO:0045893]	Chromosome [GO:0005694]; nucleus [GO:0005634]	Histone acetyltransferase activity [GO:0004402]
H9GZV1<br>A0A5F5PSP3	Ig-like domain-containing protein		Antibacterial humoral response [GO:0019731]; complement activation, classical pathway [GO:0006958]	Immunoglobulin complex, circulating [GO:0042571]	Antigen binding [GO:0003823]; immunoglobulin receptor binding [GO:0034987]
A0A0A1E3V9<br>A0A0A1E3W9<br>A0A0A1E3X3<br>A0A0A1E6Q9	Immmunoglobulin lambda light chain variable region	IGL			
Q95M34	Immunoglobulin gamma 1 heavy chain constant region	IGHC1			
H9GZV0	Immunoglobulin heavy constant epsilon	IGHE	Antibacterial humoral response [GO:0019731]; complement activation, classical pathway [GO:0006958]	Immunoglobulin complex, circulating [GO:0042571]	Antigen binding [GO:0003823]; immunoglobulin receptor binding [GO:0034987]
A0A3Q2H908<br>A0A5F5PLA4<br>H9GZQ9	Immunoglobulin heavy constant mu	IGHM	Antibacterial humoral response [GO:0019731]; complement activation, classical pathway [GO:0006958]; defense response to Gram-negative bacterium [GO:0050829]; innate immune response [GO:0045087]	Cell surface [GO:0009986]; hexameric igm immunoglobulin complex [GO:0071757]; immunoglobulin complex, circulating [GO:0042571]; membrane [GO:0016020]; pentameric igm immunoglobulin complex [GO:0071756]	Antigen binding [GO:0003823]; immunoglobulin receptor binding [GO:0034987]; peptidoglycan binding [GO:0042834]; phosphatidylcholine binding [GO:0031210]; single-stranded DNA binding [GO:0003697]
F6XAJ3	Interleukin 1 receptor accessory protein like 2	IL1RAPL2		Membrane [GO:0016020]	Hydrolase activity [GO:0016787]; interleukin-1, type II, blocking receptor activity [GO:0004910]
A0A3Q2I6F9	Keratin 82	KRT82<br>LOC1000614<br>58		Keratin filament [GO:0045095]	
F6SS14	Keratin, type I cytoskeletal 10 (Cytokeratin-10) (Keratin-10)	KRT10B	Epidermis development [GO:0008544]; epithelial cell differentiation [GO:0030855]; intermediate filament organization [GO:0045109]	Cytoskeleton [GO:0005856]; keratin filament [GO:0045095]	Structural constituent of skin epidermis [GO:0030280]
A0A3Q2GX72	MHC class I antigen 3.7	EQMCE1	Antigen processing and presentation of endogenous peptide antigen via MHC class I via ER pathway, TAP-independent [GO:0002486]; antigen processing and presentation of endogenous peptide antigen via MHC class Ib [GO:0002476]; immune response [GO:0006955]; positive regulation of T cell mediated cytotoxicity [GO:0001916]	External side of plasma membrane [GO:0009897]; extracellular space [GO:0005615]; lumenal side of endoplasmic reticulum membrane [GO:0098553]; phagocytic vesicle membrane [GO:0030670]	Peptide antigen binding [GO:0042605]; signaling receptor binding [GO:0005102]
F7D246	Mitochondrial ribosome-associated GTPase 1 (Mitochondrial GTPase 1)	MTG1	Mitochondrial translation [GO:0032543]; regulation of mitochondrial translation [GO:0070129]; regulation of respiratory system process [GO:0044065]	Mitochondrial inner membrane [GO:0005743]; mitochondrial ribosome [GO:0005761]; mitochondrion [GO:0005739]; nucleoplasm [GO:0005654]	GTP binding [GO:0005525]; gtpase activity [GO:0003924]
A0A3Q2HLE7	Muscular LMNA interacting protein	MLIP			

F6ZAE4	N-acetyltransferase 14 (putative)	NAT14			N-acetyltransferase activity [GO:0008080]
A0A3Q2HDV6	Non-specific serine/threonine protein kinase (EC 2.7.11.1)	KALRN	Phosphorylation [GO:0016310]	Cytoplasm [GO:0005737]	ATP binding [GO:0005524]; guanyl-nucleotide exchange factor activity [GO:0005085]; protein serine/threonine kinase activity [GO:0004674]
F6VKF1	Non-specific serine/threonine protein kinase (EC 2.7.11.1)	EIF2AK4	Cellular response to amino acid starvation [GO:0034198]; cellular response to cold [GO:0070417]; DNA damage checkpoint signaling [GO:0000077]; GCN2-mediated signaling [GO:0140469]; negative regulation of cytoplasmic translational initiation in response to stress [GO:1990625]	Cytoplasm [GO:0005737]; cytosol [GO:0005829]; nucleus [GO:0005634]	ATP binding [GO:0005524]; eukaryotic translation initiation factor 2alpha kinase activity [GO:0004694]
Q0PMN3	Parotid secretory protein				Lipid binding [GO:0008289]
A0A3Q2HCH7	Patatin like phospholipase domain containing 4	PNPLA4	Lipid catabolic process [GO:0016042]	Membrane [GO:0016020]	Retinyl-palmitate esterase activity [GO:0050253]; triglyceride lipase activity [GO:0004806]
A0A3Q2H7Q7	Polycystin 2 like 2, transient receptor potential cation channel	PKD2L2		Membrane [GO:0016020]	Calcium ion binding [GO:0005509]
F7D398	Proline rich 14	PRR14		Nucleoplasm [GO:0005654]	
F7BFJ1	Prothrombin (EC 3.4.21.5) (Coagulation factor II)	F2	Acute-phase response [GO:0006953]; antimicrobial humoral immune response mediated by antimicrobial peptide [GO:0061844]; cell surface receptor signaling pathway [GO:0007166]; cytolysis by host of symbiont cells [GO:0051838]; fibrinolysis [GO:0042730]; G protein-coupled receptor signaling pathway [GO:0007186]; ligand-gated ion channel signaling pathway [GO:1990806]; negative regulation of astrocyte differentiation [GO:0048712]; negative regulation of cytokine production involved in inflammatory response [GO:1900016]; negative regulation of proteolysis [GO:0045861]; neutrophil-mediated killing of gram-negative bacterium [GO:0070945]; platelet activation [GO:0030168]; positive regulation of blood coagulation [GO:0030194]; positive regulation of cell growth [GO:0030307]; positive regulation of cell population proliferation [GO:0008284]; positive regulation of collagen biosynthetic process [GO:0032967]; positive regulation of insulin secretion [GO:0032024]; positive regulation of phosphatidylinositol 3-kinase/protein kinase B signal transduction [GO:0051897]; positive regulation of phospholipase C-activating G protein-coupled receptor signaling pathway [GO:1900738]; positive regulation of protein localization to nucleus [GO:1900182]; positive regulation of protein phosphorylation [GO:0001934]; positive regulation of reactive oxygen species metabolic	Collagen-containing extracellular matrix [GO:0062023]; external side of plasma membrane [GO:0009897]; extracellular space [GO:0005615]	Calcium ion binding [GO:0005509]; heparin binding [GO:0008201]; lipopolysaccharide binding [GO:0001530]; serine-type endopeptidase activity [GO:0004252]; signaling receptor binding [GO:0005102]; thrombospondin receptor activity [GO:0070053]

			process [GO:2000379]; positive regulation of release of sequestered calcium ion into cytosol [GO:0051281]; proteolysis [GO:0006508]; regulation of cell shape [GO:0008360]; regulation of cytosolic calcium ion concentration [GO:0051480]		
F6U8V0	RNA helicase (EC 3.6.4.13) (DEAH box protein 9) (Nuclear DNA helicase II)	DHX9	DNA-templated transcription termination [GO:0006353]	Nucleus [GO:0005634]	3'-5' DNA/RNA helicase activity [GO:0033679]; ATP binding [GO:0005524]; double-stranded RNA binding [GO:0003725]; hydrolase activity [GO:0016787]; RNA helicase activity [GO:0003724]
A0A3Q2GWN9	Serotransferrin (Beta-1 metal-binding globulin) (Siderophilin)	INHCA	Intracellular iron ion homeostasis [GO:0006879]; iron ion transport [GO:0006826]	Extracellular space [GO:0005615]	Ferric iron binding [GO:0008199]
A0A5F5PW18	Serpin family F member 2	SERPINF2	Negative regulation of fibrinolysis [GO:0051918]	Extracellular space [GO:0005615]	Serine-type endopeptidase inhibitor activity [GO:0004867]
Q7M387	Serpin III				
A0A3Q2GZP8	Solute carrier family 25 member 24	SLC25A24	Transmembrane transport [GO:0055085]	Mitochondrial inner membrane [GO:0005743]	Calcium ion binding [GO:0005509]
O97678 Q9TQW8	Transferrin			Extracellular region [GO:0005576]	
A0A3Q2H472	Transporter	SLC6A1	Gamma-aminobutyric acid import [GO:0051939]; inorganic anion import across plasma membrane [GO:0098658]; neurotransmitter transport [GO:0006836]; response to inorganic substance [GO:0010035]; sodium ion import across plasma membrane [GO:0098719]; sodium ion transmembrane transport [GO:0035725]	Axon [GO:0030424]; cell surface [GO:0009986]; plasma membrane [GO:0005886]	Gamma-aminobutyric acid:sodium:chloride symporter activity [GO:0005332]
F6UL68	Transthyretin	TTR	Purine nucleobase metabolic process [GO:0006144]	Extracellular space [GO:0005615]	Hormone activity [GO:0005179]; identical protein binding [GO:0042802]; thyroid hormone binding [GO:0070324]
F6VMA0	Zinc finger and BTB domain containing 22	ZBTB22	Regulation of transcription by RNA polymerase II [GO:0006357]		DNA-binding transcription factor activity, RNA polymerase II-specific [GO:0000981]; RNA polymerase II cis-regulatory region sequence-specific DNA binding [GO:0000978]

S-Table 2. Protein information of PRP from resistant mares to post-breeding endometritis (www.uniprot.gov).

From	Protein names	Gene Names	Gene Ontology (biological process)	Gene Ontology (cellular component)	Gene Ontology (molecular function)
A0A3Q2KZ54	2-hydroxyacyl-CoA lyase 1	HACL1			Catalytic activity [GO:0003824]; magnesium ion binding [GO:0000287]; thiamine pyrophosphate binding [GO:0030976]
A0A3Q2H333	Albumin	ALB		Extracellular space [GO:0005615]	Metal ion binding [GO:0046872]
P35747	Albumin (allergen Equ c 3)	ALB	Cellular response to calcium ion starvation [GO:0072732]; cellular response to starvation [GO:0009267]; negative regulation of mitochondrial depolarization [GO:0051902]	Cytoplasm [GO:0005737]; extracellular space [GO:0005615]; protein-containing complex [GO:0032991]	DNA binding [GO:0003677]; enterobactin binding [GO:1903981]; fatty acid binding [GO:0005504]; metal ion binding [GO:0046872]; pyridoxal phosphate binding [GO:0030170]; toxic substance binding [GO:0015643]
A0A5F5PYW9<br>A0A5F5PSR8<br>A0A3Q2H846<br>F7CSL8<br>F7CYP1	Alpha-1-antitrypsin	SPI2		Extracellular space [GO:0005615]	Serine-type endopeptidase inhibitor activity [GO:0004867]
B5BV00	Alpha-1-antitrypsin	Spi2-1		Extracellular space [GO:0005615]	Serine-type endopeptidase inhibitor activity [GO:0004867]
B5BV13	Alpha-1-antitrypsin	Spi2-14		Extracellular space [GO:0005615]	Serine-type endopeptidase inhibitor activity [GO:0004867]
F6VJR6	Alpha-1-B glycoprotein	A1BG		Extracellular region [GO:0005576]; plasma membrane [GO:0005886]	
A0A5F5Q0V8<br>F7C450<br>A0A5F5PJ36	Alpha-2-HS-glycoprotein (Fetuin-A)	AHSG	Acute-phase response [GO:0006953]; negative regulation of bone mineralization [GO:0030502]; ossification [GO:0001503]; positive regulation of phagocytosis [GO:0050766]; regulation of inflammatory response [GO:0050727]	Extracellular matrix [GO:0031012]; extracellular region [GO:0005576]; extracellular space [GO:0005615]; Golgi apparatus [GO:0005794]	Cysteine-type endopeptidase inhibitor activity [GO:0004869]; endopeptidase inhibitor activity [GO:0004866]
F6R942	Alpha-2-macroglobulin	A2M		Extracellular space [GO:0005615]	Serine-type endopeptidase inhibitor activity [GO:0004867]
A0A5F5PSV7	Angiotensinogen (Serpin A8)	AGT	Regulation of systemic arterial blood pressure by renin-angiotensin [GO:0003081]; vasoconstriction [GO:0042310]	Extracellular space [GO:0005615]	Serine-type endopeptidase inhibitor activity [GO:0004867]
F7CYR1	Antithrombin-III (Serpin C1)	SERPINC1	Blood coagulation [GO:0007596]; regulation of blood coagulation [GO:0030193]	Extracellular space [GO:0005615]	Heparin binding [GO:0008201]; serine-type endopeptidase inhibitor activity [GO:0004867]
F6Z2L5	Apolipoprotein A-I (Apolipoprotein A1)	APOA1	Lipid transport [GO:0006869]; lipoprotein metabolic process [GO:0042157]; phosphatidylcholine metabolic process [GO:0046470]; regulation of intestinal cholesterol absorption [GO:0030300]	Chylomicron [GO:0042627]; high-density lipoprotein particle [GO:0034364]; multivesicular body [GO:0005771]; very-low-density lipoprotein particle [GO:0034361]	Heparin binding [GO:0008201]; high-density lipoprotein particle receptor binding [GO:0070653]; lipid binding [GO:0008289]
F6RM73	Apolipoprotein A-II (Apolipoprotein A2)	APOA2	Cholesterol homeostasis [GO:0042632]; cholesterol metabolic process [GO:0008203]; cholesterol transport [GO:0030301]; high-density lipoprotein particle assembly [GO:0034380]; high-density lipoprotein particle remodeling [GO:0034375]; lipoprotein metabolic process [GO:0042157]; low-density	Chylomicron [GO:0042627]; spherical high-density lipoprotein particle [GO:0034366]; very-low-density lipoprotein particle [GO:0034361]	Apolipoprotein receptor binding [GO:0034190]; cholesterol binding [GO:0015485]; high-density lipoprotein particle binding [GO:0008035]; high-density lipoprotein particle receptor binding [GO:0070653]; lipase inhibitor activity

			lipoprotein particle remodeling [GO:0034374]; triglyceride-rich lipoprotein particle remodeling [GO:0034370]		[GO:0055102]; phosphatidylcholine binding [GO:0031210]
A0A5F5PEE6	Apolipoprotein A-IV (Apolipoprotein A4)	APOA4	Cholesterol efflux [GO:0033344]; cholesterol homeostasis [GO:0042632]; lipoprotein metabolic process [GO:0042157]; phosphatidylcholine metabolic process [GO:0046470]; phospholipid efflux [GO:0033700]; positive regulation of fatty acid biosynthetic process [GO:0045723]; positive regulation of triglyceride catabolic process [GO:0010898]; regulation of intestinal cholesterol absorption [GO:0030300]; reverse cholesterol transport [GO:0043691]; triglyceride homeostasis [GO:0070328]; very-low-density lipoprotein particle remodeling [GO:0034372]	Chylomicron [GO:0042627]; high-density lipoprotein particle [GO:0034364]; multivesicular body [GO:0005771]; very-low-density lipoprotein particle [GO:0034361]	Heparin binding [GO:0008201]; phosphatidylcholine binding [GO:0031210]; phosphatidylcholine-sterol O-acyltransferase activator activity [GO:0060228]
F7A1W7	Apolipoprotein C2	APOC2	Chylomicron remnant clearance [GO:0034382]; high-density lipoprotein particle clearance [GO:0034384]; lipid metabolic process [GO:0006629]; lipid transport [GO:0006869]; negative regulation of very-low-density lipoprotein particle clearance [GO:0010916]; positive regulation of phospholipid catabolic process [GO:0060697]	Chylomicron [GO:0042627]; intermediate-density lipoprotein particle [GO:0034363]; low-density lipoprotein particle [GO:0034362]; spherical high-density lipoprotein particle [GO:0034366]; very-low-density lipoprotein particle [GO:0034361]	Lipid binding [GO:0008289]; lipoprotein lipase activator activity [GO:0060230]; phospholipase activator activity [GO:0016004]; phospholipase binding [GO:0043274]
F6XSF7	C4a anaphylatoxin	LOC100059239	Complement activation [GO:0006956]; complement activation, classical pathway [GO:0006958]; inflammatory response [GO:0006954]; innate immune response [GO:0045087]	Extracellular space [GO:0005615]	Endopeptidase inhibitor activity [GO:0004866]
F6PQ46	Ceruloplasmin	CP	Copper ion transport [GO:0006825]; iron ion transport [GO:0006826]	Extracellular space [GO:0005615]; plasma membrane [GO:0005886]	Copper ion binding [GO:0005507]; oxidoreductase activity [GO:0016491]; protein-folding chaperone binding [GO:0051087]
A0A3Q2GT11	Cilia and flagella associated protein 69	CFAP69			
A0A3Q2L3I3	Clusterin	CLU		Chromaffin granule [GO:0042583]; cytosol [GO:0005829]; endoplasmic reticulum [GO:0005783]; extracellular region [GO:0005576]; mitochondrial membrane [GO:0031966]; nucleus [GO:0005634]; perinuclear region of cytoplasm [GO:0048471]	
A0A3Q2HWQ6	Complement C3	C3	Complement activation [GO:0006956]; complement activation, alternative pathway [GO:0006957]; complement activation, classical pathway [GO:0006958]; inflammatory response [GO:0006954]	Extracellular space [GO:0005615]	Endopeptidase inhibitor activity [GO:0004866]
A0A3Q2GT53	C-type lectin domain family 3 member B	CLEC3B	Bone mineralization [GO:0030282]; cellular response to organic substance [GO:0071310]; cellular response to transforming growth factor	Cytoplasm [GO:0005737]; extracellular space [GO:0005615]; granular component [GO:0001652]	Calcium ion binding [GO:0005509]; heparin binding [GO:0008201]; kringle domain binding [GO:0036143]

			beta stimulus [GO:0071560]; ossification [GO:0001503]		
A0A3Q2LTU8	Ferric oxidoreductase domain-containing protein	STEAP1		Membrane [GO:0016020]	
A0A5F5PUG6	Fetuin B	FETUB			Cysteine-type endopeptidase inhibitor activity [GO:0004869]
A0A3Q2HTG2	Fibrinogen alpha chain	FGA	Blood coagulation, common pathway [GO:0072377]; cell-matrix adhesion [GO:0007160]; fibrinolysis [GO:0042730]; induction of bacterial agglutination [GO:0043152]; negative regulation of endothelial cell apoptotic process [GO:2000352]; negative regulation of extrinsic apoptotic signaling pathway via death domain receptors [GO:1902042]; plasminogen activation [GO:0031639]; platelet aggregation [GO:0070527]; positive regulation of ERK1 and ERK2 cascade [GO:0070374]; positive regulation of exocytosis [GO:0045921]; positive regulation of heterotypic cell-cell adhesion [GO:0034116]; positive regulation of peptide hormone secretion [GO:0090277]; positive regulation of protein secretion [GO:0050714]; positive regulation of vasoconstriction [GO:0045907]; protein polymerization [GO:0051258]; response to calcium ion [GO:0051592]	Endoplasmic reticulum [GO:0005783]; external side of plasma membrane [GO:0009897]; fibrinogen complex [GO:0005577]; platelet alpha granule [GO:0031091]	Cell adhesion molecule binding [GO:0050839]; signaling receptor binding [GO:0005102]; structural molecule activity [GO:0005198]
P14452	Fibrinogen alpha chain [Cleaved into: Fibrinopeptide A]	FGA	Adaptive immune response [GO:0002250]; blood coagulation [GO:0007596]; innate immune response [GO:0045087]	Extracellular region [GO:0005576]	
A0A5F5PJQ9	Fibrinogen beta chain	FGB	Platelet activation [GO:0030168]; protein polymerization [GO:0051258]	Fibrinogen complex [GO:0005577]	Signaling receptor binding [GO:0005102]
F6YW53	Geminin DNA replication inhibitor	GMNN	Animal organ morphogenesis [GO:0009887]; DNA replication preinitiation complex assembly [GO:0071163]; negative regulation of cell cycle [GO:0045786]; negative regulation of DNA replication [GO:0008156]; negative regulation of DNA-templated DNA replication [GO:2000104]; regulation of DNA-templated DNA replication initiation [GO:0030174]; regulation of mitotic cell cycle [GO:0007346]	Cytosol [GO:0005829]; nucleoplasm [GO:0005654]; nucleus [GO:0005634]; transcription repressor complex [GO:0017053]	Chromatin binding [GO:0003682]; DNA-binding transcription factor binding [GO:0140297]; histone deacetylase binding [GO:0042826]; transcription corepressor activity [GO:0003714]
A0A3Q2HBR4	Haptoglobin	LOC100067869	Acute-phase response [GO:0006953]; defense response to bacterium [GO:0042742]; proteolysis [GO:0006508]	Extracellular region [GO:0005576]	Antioxidant activity [GO:0016209]; hemoglobin binding [GO:0030492]; serine-type endopeptidase activity [GO:0004252]
F6X1I8	Hemopexin	HPX	Heme metabolic process [GO:0042168]; hemoglobin metabolic process [GO:0020027]; intracellular iron ion homeostasis [GO:0006879]; positive regulation of humoral immune response mediated by circulating immunoglobulin [GO:0002925]; positive	Extracellular space [GO:0005615]	Heme transmembrane transporter activity [GO:0015232]; metal ion binding [GO:0046872]

			regulation of immunoglobulin production [GO:0002639]; positive regulation of type II interferon-mediated signaling pathway [GO:0060335]; positive regulation of tyrosine phosphorylation of STAT protein [GO:0042531]; type II interferon-mediated signaling pathway [GO:0060333]		
A0A915VF08	Histidine-rich glycoprotein (Histidine-proline-rich glycoprotein)	hrg			Cysteine-type endopeptidase inhibitor activity [GO:0004869]
A0A3Q2I554	Histone acetyltransferase (EC 2.3.1.48)	KAT5	Positive regulation of DNA-templated transcription [GO:0045893]	Chromosome [GO:0005694]; nucleus [GO:0005634]	Histone acetyltransferase activity [GO:0004402]
H9GZV1 A0A5F5PSP3 H9GZT5	Ig-like domain-containing protein		Antibacterial humoral response [GO:0019731]; complement activation, classical pathway [GO:0006958]	Immunoglobulin complex, circulating [GO:0042571]	Antigen binding [GO:0003823]; immunoglobulin receptor binding [GO:0034987]
A0A0A1E3V9 A0A0A1E3W9 A0A0A1E3X3 A0A0A1E6Q9	Immmunoglobulin lambda light chain variable region	IGL			
Q95M34	Immunoglobulin gamma 1 heavy chain constant region	IGHC1			
H9GZV0	Immunoglobulin heavy constant epsilon	IGHE	Antibacterial humoral response [GO:0019731]; complement activation, classical pathway [GO:0006958]	Immunoglobulin complex, circulating [GO:0042571]	Antigen binding [GO:0003823]; immunoglobulin receptor binding [GO:0034987]
A0A3Q2H908 A0A5F5PLA4 H9GZQ9	Immunoglobulin heavy constant mu	IGHM	Antibacterial humoral response [GO:0019731]; complement activation, classical pathway [GO:0006958]; defense response to Gram-negative bacterium [GO:0050829]; innate immune response [GO:0045087]	Cell surface [GO:0009986]; hexameric igm immunoglobulin complex [GO:0071757]; immunoglobulin complex, circulating [GO:0042571]; membrane [GO:0016020]; pentameric igm immunoglobulin complex [GO:0071756]	Antigen binding [GO:0003823]; immunoglobulin receptor binding [GO:0034987]; peptidoglycan binding [GO:0042834]; phosphatidylcholine binding [GO:0031210]; single-stranded DNA binding [GO:0003697]
F6XAJ3	Interleukin 1 receptor accessory protein like 2	IL1RAPL2		Membrane [GO:0016020]	Hydrolase activity [GO:0016787]; interleukin-1, type II, blocking receptor activity [GO:0004910]
A0A3Q2I6F9	Keratin 82	KRT82 LOC100061458		Keratin filament [GO:0045095]	
A0A3Q2GU98	Kinesin light chain	KLC1		Cytoplasm [GO:0005737]; kinesin complex [GO:0005871]; microtubule [GO:0005874]	
A0A3Q2GX72	MHC class I antigen 3.7	EQMCE1	Antigen processing and presentation of endogenous peptide antigen via MHC class I via ER pathway, TAP-independent [GO:0002486]; antigen processing and presentation of endogenous peptide antigen via MHC class Ib [GO:0002476]; immune response [GO:0006955]; positive regulation of T cell mediated cytotoxicity [GO:0001916]	External side of plasma membrane [GO:0009897]; extracellular space [GO:0005615]; lumenal side of endoplasmic reticulum membrane [GO:0098553]; phagocytic vesicle membrane [GO:0030670]	Peptide antigen binding [GO:0042605]; signaling receptor binding [GO:0005102]
F7D246	Mitochondrial ribosome-associated GTPase 1 (Mitochondrial GTPase 1)	MTG1	Mitochondrial translation [GO:0032543]; regulation of mitochondrial translation [GO:0070129]; regulation of respiratory system process [GO:0044065]	Mitochondrial inner membrane [GO:0005743]; mitochondrial ribosome [GO:0005761]; mitochondrion [GO:0005739]; nucleoplasm [GO:0005654]	GTP binding [GO:0005525]; gtpase activity [GO:0003924]
A0A3Q2HLE7	Muscular LMNA interacting protein	MLIP			

F6VKF1	non-specific serine/threonine protein kinase (EC 2.7.11.1)	EIF2AK4	Cellular response to amino acid starvation [GO:0034198]; cellular response to cold [GO:0070417]; DNA damage checkpoint signaling [GO:0000077]; GCN2-mediated signaling [GO:0140469]; negative regulation of cytoplasmic translational initiation in response to stress [GO:1990625]	Cytoplasm [GO:0005737]; cytosol [GO:0005829]; nucleus [GO:0005634]	ATP binding [GO:0005524]; eukaryotic translation initiation factor 2alpha kinase activity [GO:0004694]
A0A3Q2H7Q7	Polycystin 2 like 2, transient receptor potential cation channel	PKD2L2		Membrane [GO:0016020]	Calcium ion binding [GO:0005509]
F6REX3	Pregnancy zone protein	LOC100061763		Extracellular space [GO:0005615]	Endopeptidase inhibitor activity [GO:0004866]; protease binding [GO:0002020]; serine-type endopeptidase inhibitor activity [GO:0004867]
A0A3Q2I7M4	Prestin (Solute carrier family 26 member 5)	SLC26A5	Inorganic anion transmembrane transport [GO:0098661]; regulation of cell shape [GO:0008360]; regulation of membrane potential [GO:0042391]; sensory perception of sound [GO:0007605]	Basolateral plasma membrane [GO:0016323]; lateral plasma membrane [GO:0016328]; lateral wall of outer hair cell [GO:0120249]	Monoatomic anion transmembrane transporter activity [GO:0008509]; protein homodimerization activity [GO:0042803]; spectrin binding [GO:0030507]
A0A3Q2HJE5	Protection of telomeres protein 1	POT1	DNA duplex unwinding [GO:0032508]; establishment of protein localization to telomere [GO:0070200]; negative regulation of telomere maintenance via telomerase [GO:0032211]; positive regulation of DNA strand elongation [GO:0060383]; positive regulation of telomere maintenance via telomerase [GO:0032212]; regulation of double-strand break repair via nonhomologous end joining [GO:2001032]; regulation of telomere maintenance via telomerase [GO:0032210]; telomere assembly [GO:0032202]; telomere capping [GO:0016233]; telomere maintenance via telomerase [GO:0007004]; telomeric D-loop disassembly [GO:0061820]	Nuclear telomere cap complex [GO:0000783]; nucleoplasm [GO:0005654]; shelterin complex [GO:0070187]	8-hydroxy-2'-deoxyguanosine DNA binding [GO:1905773]; DEAD/H-box RNA helicase binding [GO:0017151]; G-rich single-stranded DNA binding [GO:1990955]; G-rich strand telomeric DNA binding [GO:0098505]; telomerase inhibitor activity [GO:0010521]; telomeric D-loop binding [GO:0061821]
F7BFJ1	Prothrombin (EC 3.4.21.5) (Coagulation factor II)	F2	Acute-phase response [GO:0006953]; antimicrobial humoral immune response mediated by antimicrobial peptide [GO:0061844]; cell surface receptor signaling pathway [GO:0007166]; cytolysis by host of symbiont cells [GO:0051838]; fibrinolysis [GO:0042730]; G protein-coupled receptor signaling pathway [GO:0007186]; ligand-gated ion channel signaling pathway [GO:1990806]; negative regulation of astrocyte differentiation [GO:0048712]; negative regulation of cytokine production involved in inflammatory response [GO:1900016]; negative regulation of proteolysis [GO:0045861]; neutrophil-mediated killing of gram-negative bacterium [GO:0070945]; platelet activation [GO:0030168]; positive regulation of blood coagulation [GO:0030194]; positive regulation	Collagen-containing extracellular matrix [GO:0062023]; external side of plasma membrane [GO:0009897]; extracellular space [GO:0005615]	Calcium ion binding [GO:0005509]; heparin binding [GO:0008201]; lipopolysaccharide binding [GO:0001530]; serine-type endopeptidase activity [GO:0004252]; signaling receptor binding [GO:0005102]; thrombospondin receptor activity [GO:0070053]

			of cell growth [GO:0030307]; positive regulation of cell population proliferation [GO:0008284]; positive regulation of collagen biosynthetic process [GO:0032967]; positive regulation of insulin secretion [GO:0032024]; positive regulation of phosphatidylinositol 3-kinase/protein kinase B signal transduction [GO:0051897]; positive regulation of phospholipase C-activating G protein-coupled receptor signaling pathway [GO:1900738]; positive regulation of protein localization to nucleus [GO:1900182]; positive regulation of protein phosphorylation [GO:0001934]; positive regulation of reactive oxygen species metabolic process [GO:2000379]; positive regulation of release of sequestered calcium ion into cytosol [GO:0051281]; proteolysis [GO:0006508]; regulation of cell shape [GO:0008360]; regulation of cytosolic calcium ion concentration [GO:0051480]		
F6U8V0	RNA helicase (EC 3.6.4.13) (DEAH box protein 9) (Nuclear DNA helicase II)	DHX9	DNA-templated transcription termination [GO:0006353]	Nucleus [GO:0005634]	3'-5' DNA/RNA helicase activity [GO:0033679]; ATP binding [GO:0005524]; double-stranded RNA binding [GO:0003725]; hydrolase activity [GO:0016787]; RNA helicase activity [GO:0003724]
A0A3Q2GWN9	Serotransferrin (Beta-1 metal-binding globulin) (Siderophilin)	INHCA	Intracellular iron ion homeostasis [GO:0006879]; iron ion transport [GO:0006826]	Extracellular space [GO:0005615]	Ferric iron binding [GO:0008199]
A0A5F5PW18	Serpin family F member 2	SERPINF2	Negative regulation of fibrinolysis [GO:0051918]	Extracellular space [GO:0005615]	Serine-type endopeptidase inhibitor activity [GO:0004867]
F7CZW9	Serpin family G member 1	SERPING1	Negative regulation of complement activation, lectin pathway [GO:0001869]	Extracellular space [GO:0005615]	Serine-type endopeptidase inhibitor activity [GO:0004867]
Q7M387	Serpin III				
F6VCH7	SERTA domain containing 4	SERTAD4		Nucleus [GO:0005634]	
A0A3Q2GZP8	Solute carrier family 25 member 24	SLC25A24	Transmembrane transport [GO:0055085]	Mitochondrial inner membrane [GO:0005743]	Calcium ion binding [GO:0005509]
O97678 Q9TQW8	Transferrin			Extracellular region [GO:0005576]	
A0A3Q2H472	Transporter	SLC6A1	Gamma-aminobutyric acid import [GO:0051939]; inorganic anion import across plasma membrane [GO:0098658]; neurotransmitter transport [GO:0006836]; response to inorganic substance [GO:0010035]; sodium ion import across plasma membrane [GO:0098719]; sodium ion transmembrane transport [GO:0035725]	Axon [GO:0030424]; cell surface [GO:0009986]; plasma membrane [GO:0005886]	Gamma-aminobutyric acid:sodium:chloride symporter activity [GO:0005332]
F6UL68	Transthyretin	TTR	Purine nucleobase metabolic process [GO:0006144]	Extracellular space [GO:0005615]	Hormone activity [GO:0005179]; identical protein binding [GO:0042802]; thyroid hormone binding [GO:0070324]

A0A3Q2HCU6	WD repeat-containing protein 76	WDR76	DNA damage response [GO:0006974]; regulation of DNA damage checkpoint [GO:2000001]	Heterochromatin [GO:0000792]; membrane [GO:0016020]; nucleus [GO:0005634]; site of DNA damage [GO:0090734]	DNA binding [GO:0003677]; enzyme binding [GO:0019899]
F6VMA0	Zinc finger and BTB domain containing 22	ZBTB22	Regulation of transcription by RNA polymerase II [GO:0006357]		DNA-binding transcription factor activity, RNA polymerase II-specific [GO:0000981]; RNA polymerase II cis-regulatory region sequence-specific DNA binding [GO:0000978]
